# Computational methods in drug discovery

**DOI:** 10.3762/bjoc.12.267

**Published:** 2016-12-12

**Authors:** Sumudu P Leelananda, Steffen Lindert

**Affiliations:** 1 Department of Chemistry and Biochemistry, Ohio State University, Columbus, OH 43210, USA

**Keywords:** ADME, computer-aided drug design, docking, free energy, high-throughput screening, LBDD, lead optimization, machine learning, pharmacophore, QSAR, SBDD, scoring, target flexibility

## Abstract

The process for drug discovery and development is challenging, time consuming and expensive. Computer-aided drug discovery (CADD) tools can act as a virtual shortcut, assisting in the expedition of this long process and potentially reducing the cost of research and development. Today CADD has become an effective and indispensable tool in therapeutic development. The human genome project has made available a substantial amount of sequence data that can be used in various drug discovery projects. Additionally, increasing knowledge of biological structures, as well as increasing computer power have made it possible to use computational methods effectively in various phases of the drug discovery and development pipeline. The importance of in silico tools is greater than ever before and has advanced pharmaceutical research. Here we present an overview of computational methods used in different facets of drug discovery and highlight some of the recent successes. In this review, both structure-based and ligand-based drug discovery methods are discussed. Advances in virtual high-throughput screening, protein structure prediction methods, protein–ligand docking, pharmacophore modeling and QSAR techniques are reviewed.

## Introduction

Bringing a pharmaceutical drug to the market is a long term process that costs billions of dollars. In 2014, the Tufts Center for the Study of Drug Development estimated that the cost associated with developing and bringing a drug to the market has increased nearly 150% in the last decade. The cost is now estimated to be a staggering $2.6 billion dollars. The probability of a failure in the drug discovery and development pipeline is high and 90% of the drugs entering clinical trials fail to get FDA approval and reach the consumer market. Approximately 75% of the cost is due to failures that happen along the drug discovery and design pipeline [1]. Nowadays with faster high-throughput screening (HTS) experiments, which can assay thousands of molecules with robotic automation, human labor associated with screening of compounds is no longer necessary. However, HTS is still expensive and requires a lot of resources of targets and ligands. These resources are frequently not available in academic settings. Additionally, many pharmaceutical companies are now looking for ways that can avoid screening of ligands that have no possibility of showing success. Therefore, computer-aided drug discovery (CADD) tools are getting a lot of attention in the pharmaceutical industry and academia.

CADD technologies are powerful tools that can reduce the number of ligands that need to be screened in experimental assays. The most popular complementary approach to HTS is the use of virtual (i.e., in silico) HTS. Computer-aided drug discovery and design not only reduces the costs associated with drug discovery by ensuring that best possible lead compound enters animal studies, but it may also reduce the time it takes for a drug to reach the consumer market. It acts as a “virtual shortcut” in the drug discovery pipeline. CADD tools identify lead drug molecules for testing, can predict effectiveness and possible side effects, and assist in improving bioavailability of possible drug molecules. For example, in a recent study of CADD it was found that by introducing a triphenylphosphine group into the base molecule pyridazinone, it is possible to obtain inhibitors for proteasome [2]. Further, analogs have been generated using this starting structure which showed high potency. Many studies show how CADD can influence the development of novel therapeutics [3–6].

**Figure 1:**
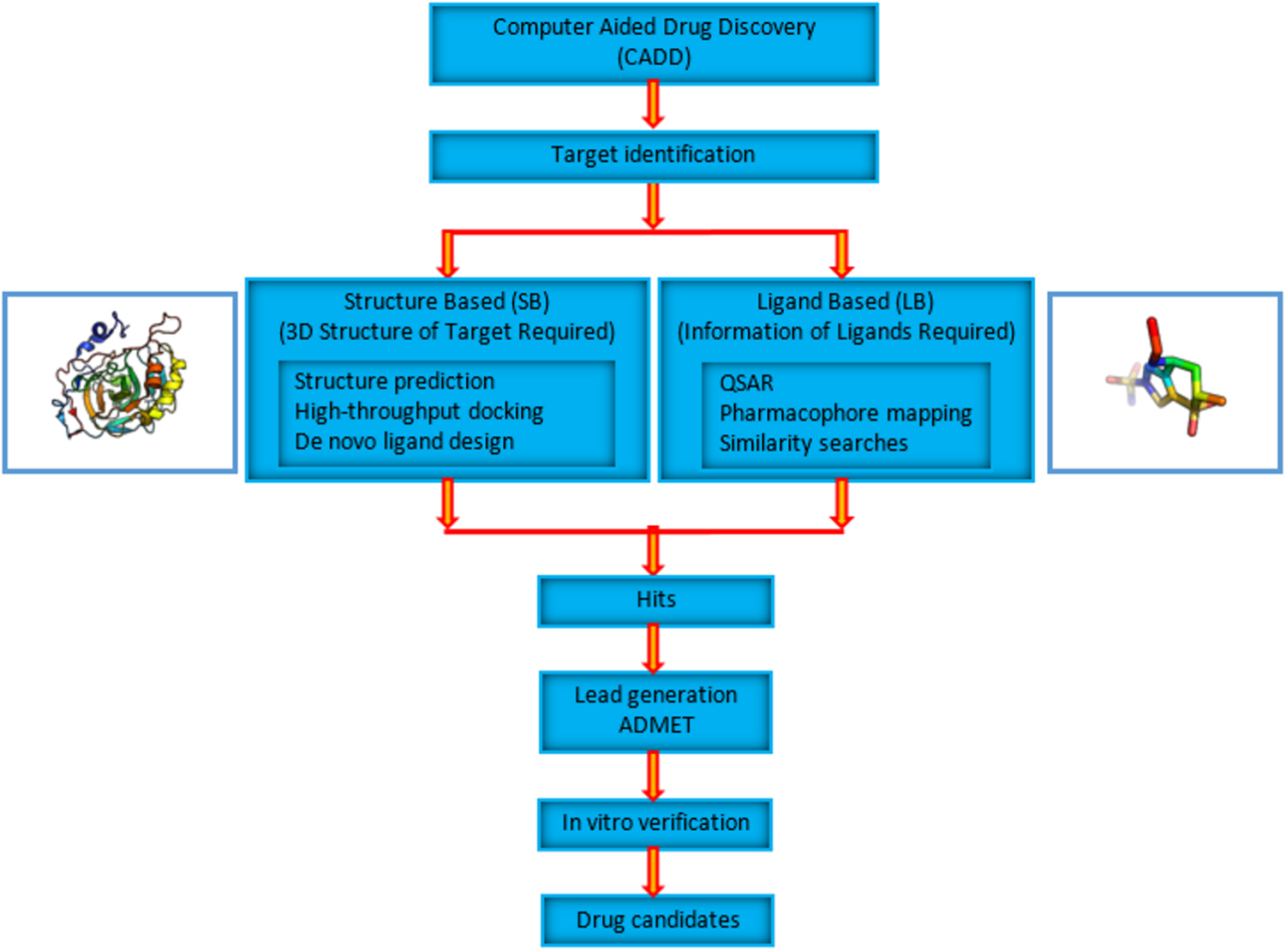
Schematic representation of a computer-aided drug discovery (CADD) pipeline. CADD methods are broadly classified into structure-based and ligand-based methods. Structure-based methods require the 3D information of the target to be known. Ligand-based methods are used when the 3D structure of the target is not known. They use information about the molecules that bind to the target of interest. Hits are identified, filtered and optimized to obtain potential drug candidates that will be experimentally tested in vitro.

CADD methods can be broadly classified into two groups, namely structure-based (SB) and ligand-based (LB) drug discovery (Figure 1). The CADD method used depends on the availability of target structure information. In order to use SBDD tools, information about target structures needs to be known. Target information is usually obtained experimentally by X-ray crystallography or NMR (nuclear magnetic resonance). When neither is available, computational methods such as homology modeling may be used to predict the three-dimensional structures of targets. Knowing the structure makes it possible to use structure-based tools such as virtual high-throughput screening and direct docking methods on targets and possible drug molecules. The affinity of molecules to targets can be evaluated by computing various estimates of binding free energies. Further filtering and optimization of possible drug molecules subsequently follow. The final selected lead molecules are tested in vitro for their activity. When the target structure is not experimentally determined or it is not possible to predict the structure using computational methods, ligand-based approaches are often used as an alternative. These methods, however, rely on the information about known active binders of the target.

CADD has played a significant role in discovering many available pharmaceutical drugs that have obtained FDA approval and reached the consumer market [7–9]. The field of CADD is rapidly improving and new methods and technologies are being developed frequently. It has immense potential and promise in the drug discovery workflow. In this review we give an overview of structure-based and ligand-based methods used in CADD, focusing on recent successes of CADD in the pharmaceutical industry. We outline structure prediction tools that are routinely used in structure-based drug discovery, widely used docking algorithms, scoring functions, virtual high-throughput screening, lead optimization and methods of assessment of ADME properties of drugs.

## Review

### Structure-based drug discovery (SBDD)

If the three-dimensional structure of a disease-related drug target is known, the most commonly used CADD techniques are structure-based. In SBDD the therapeutics are designed based on the knowledge of the target structure. Two commonly used methods in SBDD are molecular docking approaches and de novo ligand (antagonists, agonists, inhibitors, etc. of a target) design. Molecular dynamics (MD) simulations are frequently used in SBDD to give insights into not only how ligands bind with target proteins but also the pathways of interaction and to account for target flexibility. This is especially important when drug targets are membrane proteins where membrane permeability is considered to be important for drugs to be useful [10–11].

**Figure 2:**
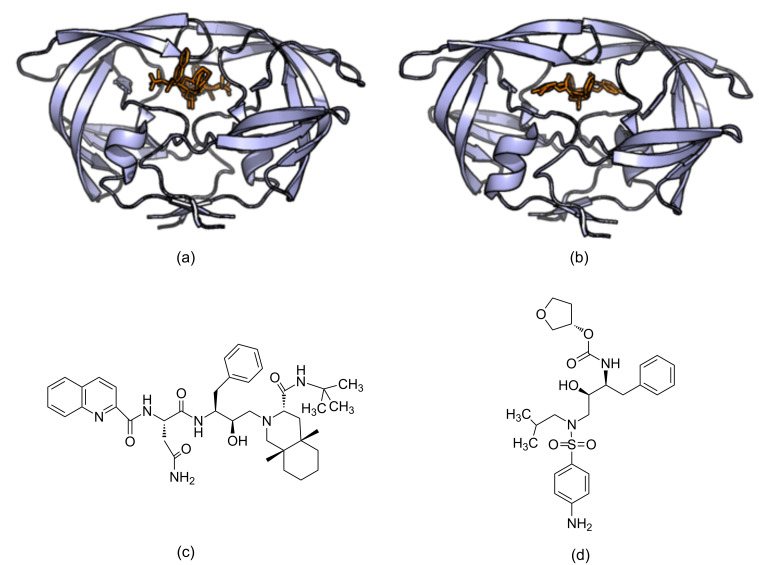
FDA approved drugs Saquinavir and Amprenavir for the treatment of HIV infections. (a) The structure of Saquinavir in complex with HIV-1 protease (3OXC) (b) the structure of Amprenavir in complex with HIV-1 protease (3NU3) (c) the molecular structure of Saquinavir and (d) the molecular structure of Amprenavir. Amprenavir and Saquinavir target HIV-1 protease and, in part, have been discovered through structure-based computer aided drug discovery methods.

**Figure 3:**
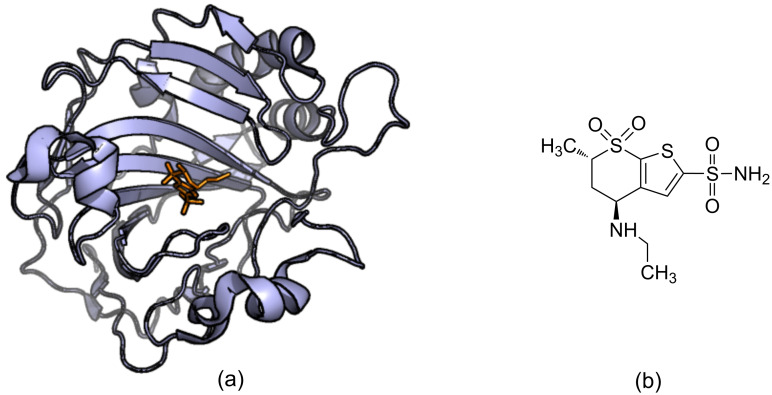
(a) The crystal structure showing the binding of Dorzolamide (orange) to carbonic anhydrase II (purple) (4M2U) (b) the structure of Dorzolamide. Dorzolamide is an FDA approved drug that targets carbonic anhydrase II to treat patience with glaucoma.

Successes have been reported for SBDD and it has contributed to many compounds reaching clinical trials and get FDA approvals to go into the market [12]. HIV-1 (Human Immunodeficiency Virus I) protease is a prime drug target for anti-AIDS therapeutics. In the early 1990s many approved HIV protease inhibitors were developed to target HIV infections using structure-based molecular docking. It was a ground breaking success at that time and made it possible for HIV infected individuals to live longer than they could have without the treatment [13–14]. Saquinavir is one of the first HIV-1 protease targeted drugs to reach the market (Figure 2a and 2c) [15]. Amprenavir is another drug that was developed to target HIV-1 protease that was also developed influenced by SBDD (Figure 2b and 2d) [16]. In another study, structure-based computational methods have been used to predict binding sites, which are important for inhibitor binding, in AmpC beta lactamase which have been experimentally verified [17]. FDA approved Dorzolamide is a carbonic anhydrase II inhibitor which is used in the treatment of glaucoma and was developed using structure-based tools (Figure 3) [7–8].

#### Protein structure determination

All structure-based methods rely on the three-dimensional target structure. The most common way to determine a protein structure is by X-ray crystallography and NMR spectroscopy. Recently, cryo-electron microscopy (cryoEM) has experienced a ‘resolution revolution’, leading to an increasing number of near-atomic resolution structures [18]. Experimental methods such as X-ray crystallography and NMR spectroscopy are associated with cost and time constraints, and are also limited by experimental challenges. X-ray crystallography is only possible if the target protein can be crystallized. Some proteins, for example membrane proteins which account for about 60% of the approved drug targets today [19], are usually difficult to crystallize, thus experimental methods are not always successful in determining their structures [20]. One of the disadvantages of NMR is that it generally is limitated to smaller proteins. Attempts are continuously being made to overcome these challenges and limitations of experimental methods [21].

SBDD methods rely on the protein structure and in the cases where the target structure is not possible to be determined by experimental methods, computational methods become useful. Determining structures from sequences using computational methods is a powerful tool that can bridge the sequence–structure gap. Importance of protein structure prediction methods and their role in drug discovery pipeline are well reviewed in literature [22–25]. Several methods have been used for protein structure prediction including homology modeling [26–27], threading approaches [28], and ab initio folding [29–30]. Several computational protein structure prediction tools that are commonly used are listed in Table 1. Large-scale genomic protein structure modeling has also been accomplished [31–32].

#### Homology (comparative) Modeling

Homology modeling is a popular computational structure prediction method for obtaining the 3D coordinates of structures. It is well known that the protein structure remains more conserved than the sequence during evolution [33–34]. The basis for homology modeling is the fact that evolutionary-related proteins often share similar structures. Knowing structures that have amino acid sequences similar to the target sequence of interest, can assist in predicting the target structure, function and even possible binding and functional sites of the structure.

In homology modeling, the first task is to find a homologous structure to the sequence of interest. To do that, the sequence is compared against a database of protein sequences where the three-dimensional structures are known [35]. NCBI Basic Local Alignment Search Tool (BLAST) is one of the most popular bioinformatics sequence alignment tools used with sequence similarity searches [36]. Once a homologous protein structure for the sequence has been identified, building the models for the target structure is done using comparative modeling algorithms [37]. The models built are evaluated and refined. Assessment of the general stereochemistry of a protein structure, such as satisfaction of bond lengths and angle restraints of generated models, is done in the model evaluation stage. Once the models are verified to be acceptable in terms of their stereochemistry, they are then evaluated using 3D profiles or scoring functions that were not used in their generation. It is generally possible to use homology modeling to predict the structure of a protein sequence that has over 40% identity to a protein of a known three-dimensional structure. When the sequence similarity drops below 30%, homology modeling is not reliable enough for structure prediction [26]. Success of homology modeling is well documented and has been continuously shown in CASP (Critical Assessment of protein Structure Prediction) which is a biannual competition aimed at determining protein structure using computational methods [38–39].

Homology modeling is commonly applied in structure-based drug discovery to predict target structures that are important in diseases [40–41]. Homology modeling of HIV protease from a distantly-related structure has been used in the design of inhibitors for this structure [42]. Similarly, M antigen structure prediction by homology modeling has given insights into function by revealing that the structures and domains are similar to fungal catalases [43]. One of the pioneering comparative modeling servers developed in the early 1990s, which is still popular today, is the SWISS-MODEL server [44–45]. MODELLER is also a popular comparative modeling program that is available as a server and also as a standalone program [46]. HHpred which is available as a server uses hidden Markov model (HMM) profiles for the detection of homology and structure prediction [47].

#### Fold recognition (threading)

Fold recognition or threading methods are used to identify proteins that do not have any sequence similarities but still have similar folds [35,48]. Fold recognition is based on the fact that over billions of years of protein structure evolution, considerable sequence divergence is observed but only small overall structural changes have occurred in protein folds [49]. Here the sequence of a known protein structure is replaced by the query sequence of the target of interest for which the structure is not known. The new “threaded” structure is then evaluated using various scoring methods [50–51]. This process is repeated for all experimentally determined 3D structures in a database and the best fit structure for the query sequence is obtained [35]. This process of identifying the best structure corresponding to the target sequence is known as fold recognition and has been used in structure-based drug discovery studies [48]. GenTHREADER is a popular fold recognition program that uses neural networks for the evaluation of the alignments [52]. MUSTER is a freely available webserver that generates sequence–template alignments for a query sequence and identifies best structure matches from the PDB [53]. In addition to sequence profile alignments, it also uses multiple structure information as well. DescFold is another webserver which employs SVM-based machine learning algorithms in protein fold recognition [54].

#### Ab initio (de novo) modeling

Ab initio or de novo modeling is employed when there is no sufficiently homologous structure to use comparative modeling. De novo protein modeling does not rely on a template structure. It models the target structure solely based on the sequence. Ab initio structure prediction implemented in Rosetta is a popular de novo structure prediction technique [55]. Here a knowledge-based scoring function is used to guide a fragment-based Monte Carlo search in conformation space. This method will generate a protein-like structure having centroid atoms to represent the side chains. Another step follows to refine this centroid-based structure using an all-atom refinement function in order to relax the structure. Rosetta protein structure prediction methods have shown successes in CASP experiments [56]. Ab initio structure prediction server QUARK, developed by the Zhang group has also shown great success in recent CASP experiments [57]. QUARK uses atomic knowledge-based potential functions and models are built from small residue fragments by replica exchange Monte Carlo simulations. In both CASP9 and CASP10, QUARK was the number one ranked server in the template free modeling category outperforming the Rosetta server though Rosetta remains to be one of the most popular methods of ab initio structure prediction. Many other ab initio structure prediction software packages have been developed in the last three decades and some of the popular ones are listed in Table 1.

**Table 1:** Some of the popular structure prediction tools, methods of prediction and their availability.

Tool	Method	Availability	Citation

**Homology**			
3D-JIGSAW	Fragment-based assembly	server	[58]
MODELLER	Satisfaction of spatial restraints	server/download	[46]
HHpred	Pairwise comparison of profile HMMs	server/download	[47]
RaptorX	Single/multi-template threading, alignment quality prediction	server	[59–60]
Swiss model	Fragment-based assembly and local similarity	server	[44]
Phyre2	Advanced remote homology detection, effect of amino acid variants	server	[61–62]
**Fold recognition**			
MUSTER	Profile-profile alignment with multiple structural information	server	[53]
GenTHREADER	Sequence alignment, threading evaluation by neural networks	server/download	[52]
I-TASSER	Iterative template fragment assembly	server/download	[63]
**Ab initio**			
QUARK	Replica-exchange MC and optimized knowledge-based force field	server	[57]
Rosetta/Robetta	Fragment assembly, simulated annealing	server/download	[55,64–65]
I-TASSER	Fragment assembly	server/download	[63]
CABS-FOLD	User provided distance restraints from sparse experimental data	server	[66]
EVfold	Calculate evolutionary variation by co-evolved residue pairs	server	[67]

#### De novo modeling with sparse experimental restraints

Ab initio prediction of protein structures starting from the sequence is challenging and success is often limited to only small proteins [65]. However, ab initio structure prediction can be guided by the use of sparse experimental data [68]. NMR information has been used in many studies to intelligently guide protein structure prediction [69–71]. NMR Nuclear Overhauser Effect (NOE) data and chemical shifts have been used in combination with Rosetta ab initio structure prediction to obtain better protein structure predictions [72]. Freely available CABS-FOLD uses a reduced representation approach and lets the user provide experimental distant restraints in ab initio structure prediction [66]. This method was successful when tested in CASP6 for targets for which the necessary NMR data already existed [69]. NMR data is not the only form of experimental data that can be used in ab initio structure prediction. With the EM-Fold method it is possible to obtain atomic level protein structures using only the protein sequence information and medium-resolution electron density maps [73–74]. Sparse electron paramagnetic resonance (EPR) spectroscopy data has also been used in high-resolution de novo structure prediction [75–77].

#### Protein and small molecule databases

Information about drug molecules and target structures is critical in using SBDD tools and many repositories collect and store such information about small molecules and target proteins. PubChem, a small molecule repository is available through NIH which contains millions of biologically relevant small molecules [78]. ZINC is a virtual high-throughput screening compound library which is a free public resource [79–80]. This database contains over 35 million molecules that are purchasable and are available in 3D formats. These molecules have all been pre-processed and are ready for docking. DrugBank has about 5000 small molecules and more than 3000 of these are experimental drugs [81]. There are over 800 compounds in DrugBank that are FDA approved.

The Protein Databank (PDB), which was first introduced in 1970s, is a global resource that contains a wealth of 3D information about experimentally determined biological macromolecules [82–83]. The structures in the PDB are individual macromolecules, protein–DNA/RNA or protein–ligand complexes. Experimental methods used in structure determination are mostly X-ray crystallography and NMR spectroscopy. As of 2016, the PDB databank contains around 120,000 biological macromolecular structures that have been deposited. It has structural information on over 20,000 bound ligand molecules as well. Swiss-Prot is a database which has non-redundant protein sequences which are manually annotated to contain descriptions such as functional information of protein sequences and post-translational modifications [84]. PDB and Swiss-Prot are both general purpose biological databases.

There are other databases that contain specific biological information as well. The BIND database contains protein complex information and biomolecular interactions [85]. BindingDB contains measured binding affinity information of proteins that are considered to be targets for drugs [86]. This database contains over one million binding data points.

#### Binding pocket identification and volume calculation

Once a protein’s three-dimensional structure is known, finding binding pockets on that protein is an important next step in structure-based drug discovery. It can give indications of where small molecules can bind to target structures, which are associated with diseases, contributing to increase or decrease of target activity. Binding sites in target proteins can be experimentally determined; for example using site-directed mutagenesis or X-ray crystallography. There are also a variety of computational binding pocket identifying algorithms available for the drug discovery scientific community [87].

**Figure 4:**
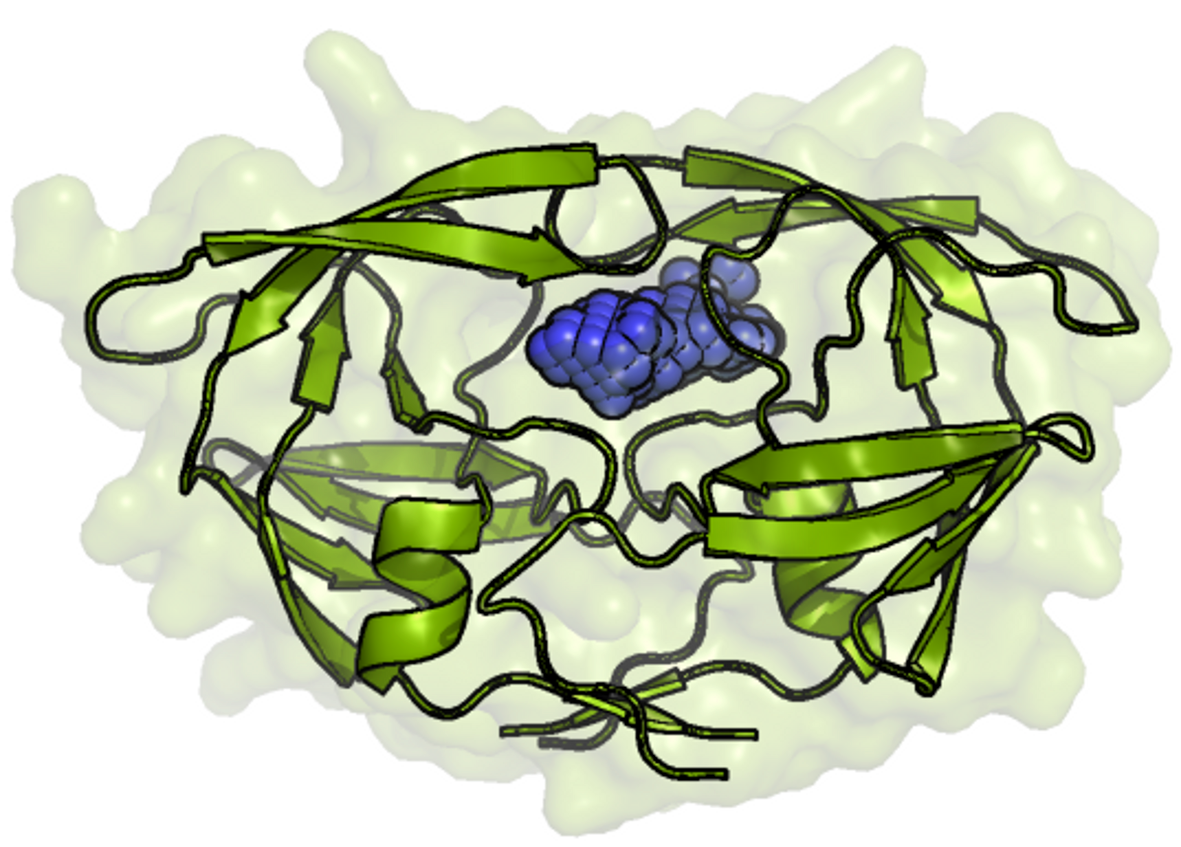
The best ligand binding site identified by SiteHound in HIV-1 protease. The ligand binding pocket is shown in blue spheres and is the known inhibitor binding site of HIV-1 protease.

Binding pocket predicting algorithms can be grouped into two broad categories; geometry-based and energy-based methods. In many cases the binding pocket is considerably larger than all the other pockets in a target and it has been found that in 83% of enzymes that are single chain, the ligands bind to the largest pocket in the enzyme [88]. According to this finding, the binding pockets of a target could be predicted by the geometry of the target. Therefore the size of the pocket is important for function as well. One of the geometry-based binding site identification servers is 3V [89]. Even though for some cases the largest pocket or cleft of a protein is its binding pocket, it is not necessarily true for all target proteins. Energy-based methods have been developed to address this issue and have shown more success than geometry-based methods [90]. In Q-SiteFinder a van der Waals probe is used and the interaction energy between the probe and the protein is found in order to identify binding sites of the protein [91]. The SiteHound program is another energy-based method that uses two kinds of probes; a carbon probe and a phosphate probe which are used to identify the binding sites for drug-like molecules and phosphorylated ligands (such as ATP) respectively [92]. The best ligand binding site identified in HIV-1 protease by SiteHound is shown in Figure 4. This ligand binding site is the known inhibitor binding site in HIV-1 protease. Another energy-based method, FTMAP, uses 16 probes in identifying hot spots in structures and was more recently extended to include any user provided small molecule as an additional probe [93–94]. Many other binding pocket finding programs exist. PEP-SiteFinder [95], SiteMap available through Schrodinger [96] and MolSite [97] are a few of these programs.

When the binding pocket of a target is known one significant characteristic to be calculated is its binding pocket volume. With this information elimination of ligands that are too bulky to fit in the pocket can be done during the lead identification process. One algorithm that calculates the volume of a binding pocket is POVME (POcket Volume MEasurer) [98]. McVol is another standalone program that can identify and calculate the volume binding cavities in protein structures by using a Monte Carlo algorithm [99].

#### Scoring functions used in docking

In molecular docking, how well a drug binds to its target is determined by the binding affinity prediction of the pose. This is done by scoring. Scoring is used to evaluate and rank the target–ligand complexes predicted by docking algorithms. Scoring functions are used in SBDD for scoring and evaluating protein–ligand interactions [100–101]. The scoring function used by docking algorithms is a crucial part of the algorithm. It is used in the exploration of the binding space of the ligand and also in the evaluation of target–ligand complexes in molecular docking. The scoring functions can be categorized into knowledge-based [102–103], force-field based [104–105], empirical [106–107] and consensus [108–109]. These will be discussed below. The accuracy of different scoring functions has been evaluated in the literature [110–113]. These comparative studies that evaluate docking method scoring functions use evaluation criteria such as binding pose, binding affinity and ranking of true binders [114]. Wang et al. evaluated the performance of fourteen different scoring functions using 800 protein–ligand complexes in the PDBbind database [113]. The performance was evaluated by the predicted binding affinities of protein–ligand complexes by different scoring functions. According to this study X-Score, DrugScore and ChemScore were among the best performing scoring functions. Ferrara et al. used nine scoring functions and assessed the performance of these functions using 189 protein–ligand complexes [110]. They found that ChemScore shows the best correlation for experimental binding energies and predicted binding scores. In another study done by Marsden et al., calculated binding free energies with knowledge-based potential function Bleep agreed best with experimental binding constants [115].

#### Knowledge-based scoring functions

Knowledge-based scoring functions are statistical potentials and are derived from experimentally determined protein–ligand information. The frequency of occurrence of interactions of a large number of target–ligand complexes are used to generate these potentials. The basis of these potentials is the Boltzmann distribution. The frequency of occurrence of atom pairs is converted into a potential using Boltzmann’s distribution of states. Since these potentials use target–ligand complex data already available, they are highly dependent on the dataset used to create them. DrugScore uses knowledge-based potential functions to predict binding affinity [116]. Other knowledge-based scoring functions include the PMF (Potential of Mean Force) [117] and Bleep [118].

#### Force-field-based scoring functions

Force-field based energy functions are developed using classical molecular mechanics. Electrostatic (coulombic) interactions and van der Waals interactions (Lennard-Jones potential) contribute to the interaction energy between a target–ligand complex. Two of the most widely used molecular mechanical force-fields are CHARMM [119] (Chemistry at HARvard Macromolecular Mechanics) and AMBER [120] (Assisted Model Building and Energy Refinement) which have been built mainly for molecular dynamics simulations. The molecular docking program DOCK [121] uses force-field based scoring functions derived from molecular dynamics force-field AMBER.

#### Empirical scoring functions

Empirical scoring functions are obtained by using data from experimentally determined structures and fitting this information to parameters. The idea here is that the binding free energy is calculated as the weighted sum of terms that are uncorrelated. These terms can be the number of hydrogen bonds, hydrophobic effect, and different types of contacts and their types etc. Regression analysis is usually done to obtain weights of the terms using experimental target–ligand complexes with known binding free energy data [122]. Unlike knowledge-based scoring functions, which are obtained by directly converting frequency of occurrence of different interactions into potentials using Boltzmann principle, these functions take into account multiple terms or contributions and find the best weights for each term using regression analyses. The HYDE scoring function is an empirical energy function which is a part of BioSolveIT tools [123]. Here the binding energy of a target–ligand complex is solely estimated by a hydrogen bond term and a dehydration energy term. ChemScore [122] and SCORE [124] are two other scoring functions that are also empirical.

#### Consensus-based scoring functions

Current scoring functions are not perfect and no one scoring function can do well in every docking complex studied. Consensus scoring was introduced to combine different scoring functions in the hope that it can balance out errors and improve accuracy [125]. Consensus scoring function X-CSCORE [126] was developed by combining three different empirical scoring functions, namely Bohm’s scoring function [127], SCORE and ChemScore. Another example of consensus scoring is MultiScore [128]. This score function is a combination of eight different scoring functions and have shown improved protein–ligand binding affinities.

#### Protein–ligand docking algorithms

In docking, predictions are made on how intermolecular complexes are formed between a target and a ligand. These algorithms search for the best target–ligand poses with the right conformational state and relative orientation. The algorithms also crudely estimate the binding affinities of the target–ligand complexes in terms of scoring. The docking algorithms therefore comprise a search algorithm that searches the conformational space to find docking poses and a scoring function to predict the affinity of the ligand in that pose. Computationally docking a target structure to a molecule is a challenging process. Even when target flexibility is ignored there are still a huge number of ways a molecule can be docked. The total number of possible modes increases exponentially as the size of the two docked molecules increases. Therefore efficient search methods that are fast and effective, and reliable scoring functions are critical components of docking algorithms.

Once a target protein structure is known and a potential drug binding site has been identified, small molecules that bind to this site need to be determined. In drug discovery, docking algorithms are used to find the best fit between a target and a small molecule drug. Docking algorithms require a target protein structure and a library of small molecules. The target protein structure is usually determined using experimental methods such as X-ray crystallography and NMR, or else it is computationally modeled. Molecular docking aims to predict the binding mode and binding affinity of a protein–ligand complex. A library of small molecules is virtually placed (docked) into the desired protein–target binding site and thousands of possible poses of binding are obtained and evaluated. The pose which is scored with the lowest energy is predicted to be the best possible binding mode. The models are evaluated using a scoring function and the poses are ranked and a group of high ranking compounds are chosen for the next step of experimental verification.

One of the very first studies that developed algorithms to evaluate docking poses by looking at steric overlaps was published in the 1980s [129]. Ever since then many docking algorithms have been developed [130–135]. Popular molecular docking programs include Glide [131], Fred [136], AutoDock3 [137], AutoDock Vina [134], GOLD [138] and FlexX [139]. AutoDock3 uses an empirical scoring function that has five terms. These terms are weighted with experimental target–ligand data. It can model side chain flexibility of the target molecule. AutoDock Vina is the new generation of AutoDock. The scoring function used in AutoDock Vina is a hybrid scoring function that combines knowledge-based and empirical scoring functions [134]. GOLD uses a force-field based scoring function and allows the ligand to be fully flexible. It allows target side chain flexibility to be taken into account. FlexX is an incremental fragment-based docking algorithm where the conformational space sampling is done using a tree-search method. It is an ensemble method that can incorporate target structure flexibility. The scoring function is a modified version of empirical Bohm’s scoring. Glide is a highly popular docking algorithm that uses an empirical scoring function [131]. Fred, by OpenEye Scientific, finds protein–ligand docking poses by using a non-stochastic exhaustive method. It uses filters that take shape complementarity into account and the top scoring poses selected are further optimized [136]. Docking algorithms are discussed in detail in reviews [140–142] and comparative assessment of algorithms have been done [112,143–145]. Zhou et al. evaluated the performance of several flexible docking algorithms by calculating enrichment factors for a set of pharmaceutical target–drug complexes and found that Glide XP was superior to other methods tested [144]. The study done by Perola et al. shows that Glide is superior to other methods tested for the prediction of binding poses but virtual screening is mostly target-dependent [112].

The best docking algorithm should be the one with the best scoring function and the best searching algorithm. The performance of various docking algorithms has been evaluated and they are able to generate docked ligand conformations that are similar to experimental complexes [146]. Compared to co-crystallized X-ray structures of target–ligand complexes docking results can sometimes even predict poses with RMSDs of less than 1 Å [147]. Measuring RMSD (root mean square deviation) is the most common way to compare the structural similarity between two superimposed structures. RMSD is given by:


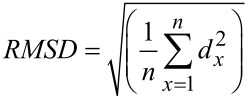


where *n* is the number of atom pairs and *d*_x_ is the distance between the two atoms in the *x*^th^ atom pair.

**Figure 5:**
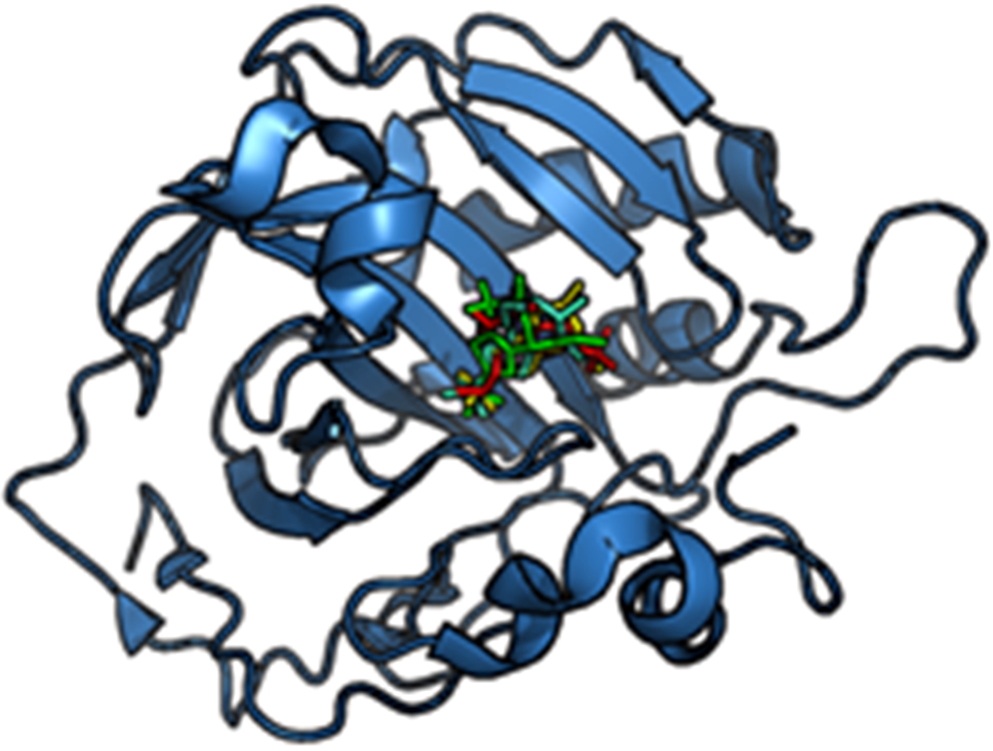
Binding mode prediction. The known inhibitor Dorzolamide is docked into Carbonic anhydrase II crystal structure (4M2U) (blue) using AutoDock Vina. Four binding poses predicted are shown in green, cyan, red and yellow. The molecular structure of Dorzolamide is shown in Figure 3b.

However, it is important to note that no single docking method performs well for all targets and the quality of docking results is highly dependent on the ligand and the binding site of interest [148–150]. The best four binding poses predicted for the known inhibitor Dorzolamide binding to carbonic anhydrase II obtained by AutoDock Vina are shown in Figure 5.

#### Preprocessing of target and ligands

Target and ligand preparation steps are crucial and are often done before docking is performed to ensure good screening results [151]. In experimental methods such as X-ray crystallography the hydrogen atoms of structures are not generally present. However, the presence of these atoms and the locations of these bonds are important for molecule docking algorithms. Additionally, the target protein structures, if used without preprocessing, can give rise to potential issues due to missing residues, atom clashes, crystallographic waters and alternate locations. In target preprocessing, missing atoms such as hydrogen are added and atomic clashes are removed. The same is true for the ligands that are used. During ligand preprocessing, ligand three-dimensional geometries are predicted. All possible ionization, stereoisomeric and tautomer states are assigned [152]. The protonation states of structures are also important in prediction of docking poses because protonation states affect how ligands bind to the binding site [100]. Optimizing protonation states of binding pockets and also positions of polar hydrogens can lead to identifying the most native-like docking poses.

SPORES is one program that is used for the prepossessing of proteins for protein–ligand docking. It can generate different protonated states, tautomeric states and stereoisomers for protein structures [152]. LigPrep from the Schrodinger Suite [153] allows to obtain all-atom 3D structures of ligands. It is available through the maestro interface or by command line. A web-based ligand topology generating server, PRODRG, can generate 3D coordinates for ligands that are of equal or better quality than other methods [154].

#### De novo ligand design

By using fragment-based de novo ligand design it is possible to assemble molecules that are drug-like with much less search space having to be explored. In some cases, de novo drug design is less successful in generating drug candidates compared to other methods such as high-throughput virtual screening methods for large databases. One limitation of this approach can be attributed to its high complexity. When a high-resolution target structure is available, ligand growing programs such as biochemical and organic model builder (BOMB) can be used to design ligands that bind to the target without using ligand databases [155–156]. Using BOMB it is possible to grow molecules by adding substituents into a core structure. It has been possible to design inhibitors for Escherichia coli RNS polymerase using the de novo drug design program SPROUTS [157]. In another study that used the SPROUT program, novel inhibitors were developed for Enterococcus faecium ligase VanA using hydroxyethylamine as the base template structure [158]. It is generally necessary to synthesize molecules that are obtained by de novo drug design. Whereas when using virtual screening methods, since the screening is usually done with databases of commercially available molecules, it is possible to purchase these molecules without the need to synthesize them. LigMerge is another novel algorithm that can generate novel ligands for drug targets [159]. It uses known ligands of a target and generates models with similar chemical features by finding the maximum common substructure of known ligands. Chemical groups of superimposed ligands are attached to the common substructure. This produces different molecules that have features of the known ligands. The algorithm is able to identify novel ligands for several known drug targets that have predicted affinities higher than their known binders. The AutoGrow software is a drug molecule optimizing program. It can be used to optimize ligands according to various properties and binding affinities and is available to download [160]. If two fragments bind to two non-overlapping nearby sites on a target protein, these fragments can be joined to obtain a possible new drug molecule. In the SILCS (site identification by ligand competitive saturation) method, molecular dynamics simulations are used to identify fragments that bind to a target [161–162]. SILCS uses explicit molecular dynamics simulations where the target molecule is simulated in an aqueous solution that contains different fragments. Using multiple simulations SILCS determines high probability binding areas of the target for the different fragments which can be used in fragment-based drug design. Another de novo ligand design program is LigBuilder which is available for download [163–164]. Here the ligands are either grown or linked by user’s choice, and an empirical scoring function is used to estimate binding affinities.

#### Structure-based virtual high-throughput screening (VHTS)

Structure-based virtual high-throughput screening (VHTS) is large scale in silico screening of drug molecules in databases of small molecule compounds for a target of interest. Here a target is “screened” against a library of drug-like molecules and binding affinities of the ligands to the target are estimated using the scoring functions described previously. In addition to finding the best docking pose VHTS also ranks docking results according to their predicted affinities for the entire database. Since large databases are screened, it is important that the target–ligand docking algorithms used in VHTS are both fast and sufficiently accurate, to be able to identify a subset of possible drug compounds. Small molecules that are predicted to bind to the target with high affinity can be identified. These “hits” are then generally further optimized and subsequently tested experimentally. With improving computational resources and parallel processing cluster availability, it is now possible to screen millions of compounds within a matter of hours or days. Because of VHTS it is possible to experimentally test a rather small number of molecules. Testing thousands of available compounds in databases experimentally may no longer be necessary.

Structure-based high-throughput screening has been used to identify inhibitors of protein kinase CK2 targeting its ATP binding site [165]. CK2 is an important target in developing antitumor drugs. About 400,000 compounds have been screened, from which 12 hits were selected for evaluations using in vitro assays. Out of these hits a novel drug was identified which was able to inhibit CK2 enzymatic activity with an IC_50_ of 80 nM. At the time it was discovered, this drug was considered one of the most potent drugs for a protein kinase. In another study, VHTS was used to show proliferator-activated target agonistic behavior with Sulfonylureas and Glinides binding [166]. This finding has implications in the treatment of type-2 diabetes and it was also confirmed by experimental assays. Recently, virtual screening has been used to find anitiviral inhibitors that target the Ebola virus. This study found a lead candidate from the TCM database that shows a decrease in activity for the protein encoded by the virus [3]. This promising candidate also shows good pharmacokinetic properties. VHTS was also used to find a novel quinolinol that binds to MDM2 in a fashion mimicking the binding of p53 and inhibit the MDM2-p53 interaction [167]. This inhibition can activate p53 in cancer cells. There are many more examples where VHTS has been used in drug discovery studies [168–172].

#### Target flexibility in molecular docking

Experimental methods such as X-ray crystallography represent proteins as static structures. However proteins are dynamic systems and show internal motions. The functionality of proteins is governed by their internal dynamics. In order to explain their function it is important to understand their dynamic characteristics [173]. In the first molecular docking attempts in the 1980s, the rigidity of the target protein was always assumed [129]. This has not changed significantly until recently; some newer docking algorithms can account for target flexibility. It is important to account for target protein flexibility because protein structures are dynamic in nature and their structures change upon binding of drug molecules. These changes may involve overall backbone structure rearrangements or they can be subtle where only the side chains near the ligand binding site change to accommodate the bound ligand. However, this dynamic nature of targets is frequently ignored and protein flexibility is underrepresented in CADD. In conventional docking algorithms the target is held rigid while the ligand molecule is generally assumed to be flexible. This rigid body docking of ligands to the target is not realistic and can give misleading results because targets are actually able to freely undergo side chain and backbone movements as a result of ligand binding by an induced fit mechanism [174]. Two approaches that can be taken to account target flexibility are induced fit docking methods and ensemble-based screening methods.

In induced fit docking the target protein structures are modeled as flexible, not rigid. They are able to accommodate induced fit that is caused by the ligand molecule binding to it. Schrodinger has introduced induced fit docking protocols through Glide [174]. RosettaLigand also accounts for target flexibility and shows success in predicting target–ligand poses. All residue side chain flexibility of the ligand binding pocket and target backbone flexibility are taken into account by RosettaLigand. This method uses a Monte-Carlo-based minimization algorithm and the Rosetta full-atom energy function [175].

**Figure 6:**
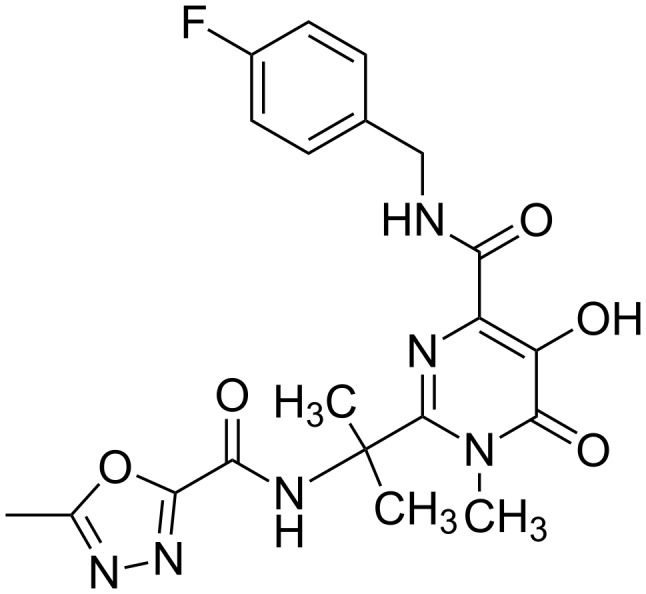
The molecular structure of Raltegravir. Raltegravir is an FDA approved drug used in the treatment of HIV infection.

Ensemble-based docking is an alternative method to induced fit docking. With ensemble-based screening methods there is no need to choose flexible residues of interest to binding [176–180]. The relaxed complex scheme (RCS) method uses structure dynamics and docking algorithms in combination to account for target flexibility [181–182]. One successful application of RCS was reported for HIV integrase. MD simulations that were performed with the holo-structure of HIV integrase bound to a known ligand showed signs of a novel binding pocket opening in close proximity to its active site [183]. RCS ligand docking showed that this binding site is a possible binding pocket for drug molecules. This finding paved the way for the development of raltegravir to treat HIV infection which was later approved by the FDA (Figure 6) [184–185]. These ensemble-based screens use an experimentally determined starting target structure and use various methods to generate target structure ensembles. These methods include molecular dynamics simulations [186], Monte Carlo simulations [187], enhanced sampling [177] or just simply experimental ensembles from NMR or multiple crystal structures, to generate an ensemble of conformations based on the starting target structure. By doing so, conformations of the target structure that are relevant to the biological function which are not accessible by experimental structure determination, can be obtained. The trajectories can be clustered to obtain a set of representative conformations. The difference here is that instead of using one structure, an ensemble of structures is used in docking. Due to the range of conformations generated by these methods a more representative set of small molecules can now bind to the ensemble [6,188–189].

#### Enhanced sampling methods

Ensemble-based methods which are typically employed to account for target flexibility use enhanced sampling methods. One of the tools that is extensively and routinely used to understand protein motions and conformational space that is accessible for protein structures is molecular dynamics (MD) simulations [190]. The most widely used molecular dynamics software packages are NAMD [191], GROMACS [192] and AMBER [120]. The typical time-scale of a molecular dynamics simulation is in the order of nanoseconds to microseconds. However to capture biologically important conformational transitions, frequently it is important to probe dynamics in the order of milliseconds. Simulating on that timescale makes it possible to overcome high-energy barriers in some important biological transitions. With conventional all-atom MD simulations and typically available computational resources this can be a very time consuming process. Millisecond-scale MD simulations are possible with high speed supercomputers, although most computational scientists do not have access to such powerful machines [193]. This is considered a major limitation of MD. It may take months to complete a one microsecond molecular dynamics run on a system having around 25000 atoms on 24 processors [194]. Enhanced sampling methods have been introduced to address this issue [176,195]. With enhanced sampling methods it is possible to find conformational states that are relevant to the function of proteins that are not explored in conventional MD. Enhanced sampling methods introduce a bias on the system being simulated. Several methods of enhanced sampling are introduced in literature, including: accelerated molecular dynamics [196–197], metadynamics [198–199], umbrella sampling [200] and temperature-accelerated molecular dynamics [201–202].

Accelerated molecular dynamics (aMD) simulations reduce the energy barrier of wells or in other words raise the energies of the wells that are below a certain threshold energy [203]. This leaves the high-energy states above the cutoff unaffected. When the original energy of the system is below the calculated energy, an additional potential term is added (a boost potential), thereby allowing energy barriers to be smaller. This makes it possible for the system to access conformations which are not accessible without the energy barrier reduction [203–205].

In metadynamics a history-dependent bias potential (which is a function of a set of collective variables) or a force is added to the Hamiltonian of the system to accelerate the system in consideration by pushing it from the local energy minimum [199]. It is important that the collective variables used can describe the initial, final and intermediates states. Commonly used collective variables are interatomic angles, dihedrals and distances. By doing so, it is possible to sample rare events that are otherwise not sampled by conventional MD. Finding the set of collective variables however is challenging especially when the simulated biological system is more complex. Recently, induced fit docking has been coupled with metadynamics to predict protein–ligand complexes in a reliable way. By incorporating metadynamics with induced fit methods, the predictive power of these methods can be enhanced without requiring too much computational resources [4].

The umbrella sampling technique is used to calculate free energy differences in systems [200]. An additional energy term or a bias potential is introduced to the system along a reaction coordinate. This bias potential can then drive the system from the reactant state to the product state. Each of the intermediate states is simulated by MD. Most of the time, for reasons of simplicity, bias potentials are applied as harmonic potentials [200,206].

In temperature-accelerated MD, the system simulation is done at a high enough temperature which makes it possible to accelerate the sampling. Temperature accelerated MD has been used in the study of ligand dissociation from the inducer binding pocket in the Lac repressor protein [202]. By using this method, it was possible to sample the dissociation trajectories in a relatively short period of time to capture the ligand dissociation. The replica exchange method runs a number of independent replicas in different ensembles of the systems at different temperatures and allows exchange of replica coordinates to take place between these ensembles [207]. This method can also enhance sampling in cases where the energy landscape of a system has many minima and where it is not possible to cross the barriers between them during standard simulation times.

#### Rigorous binding free energy calculations

**Figure 7:**
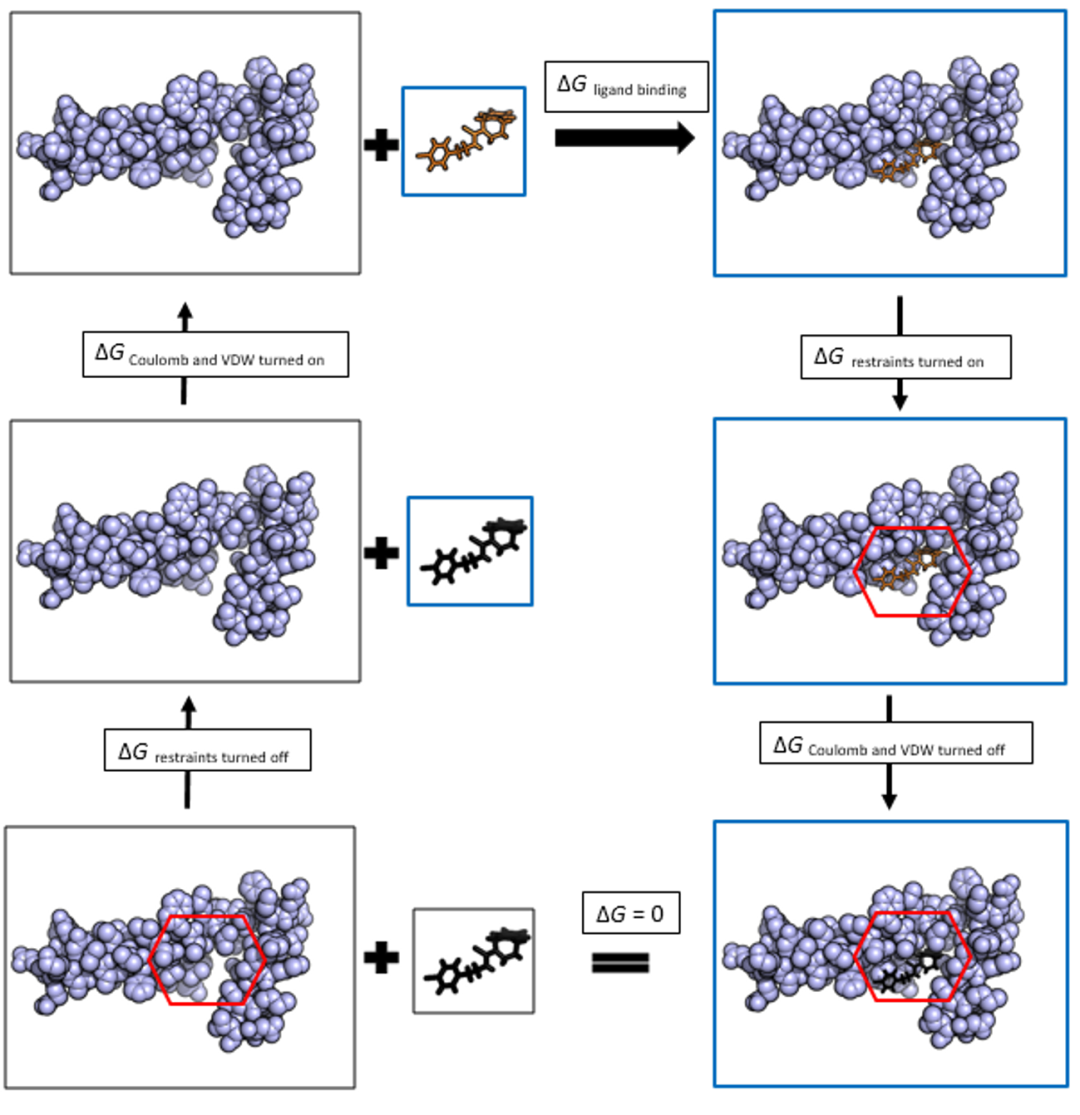
An example alchemical thermodynamic cycle for a protein–ligand binding free energy calculation. The protein is shown in blue spheres. The ligand, depicted in solid black, indicates there are no coulombic or van der Waals (VDW) interactions with the environment. The ligand, depicted in solid orange, indicates there are coulombic and VDW interactions with its environment. The systems that are subjected to simulations in each cycle are highlighted in blue boxes. All simulations are run in a water environment. The first step is to add restraints between ligand and the protein in order to keep the ligand confined to the binding pocket and to avoid the ligand leaving the pocket when its interactions are removed. The systems with restraints turned on are indicated by red hexagons. In the next step the coulombic and VDW interactions of the ligand are removed. This step is followed by the removal of the restraints applied to the ligand. Next the coulombic and VDW interactions of the ligand are turned on such that the ligand is in contact with solvent. Summing up the free energy changes along the thermodynamic cycle would give the protein–ligand binding free energy.

Rigorous binding free energy calculations can be used to more precisely estimate the binding affinity of target–ligand complexes and these affinities can be used to rank the fit of drug molecules for a particular target. Binding affinities can be used to infer how drug binding will be affected by target mutations [208]. The potency of a drug is assumed to be directly related to the target–drug molecule binding affinity. Therefore it is important to be able to accurately predict the target–ligand binding affinity [209]. Currently the most accurate approaches to calculate binding free energies are rigorous approaches [210]. Monte Carlo algorithms and molecular dynamics simulations are used for generating ensemble averages to model complexes in the presence of explicit water molecules using classical force-fields. Two rigorous binding free energy approaches are the free energy perturbation (FEP) methods and thermodynamic integration (TI) methods [211–215]. These methods are much more accurate than virtual screening. Both of these methods are rigorous alchemical (non-physical) transformations, where the transformation happens via an alchemical pathway of states in a thermodynamic cycle (Figure 7). By using these intermediate states, the starting state of a biological system can be transformed into another state. Turning off atom charges is one example of an intermediate pathway of an alchemical thermodynamic cycle. The binding free energy is computed as a sum of all the steps in the cycle from unbound to bound.

Free energy perturbation is one of the most popular molecular simulation-based free energy calculation methods [213]. It was first introduced by Zwanzig in the 1950s [216]. This method uses statistical mechanics as well as molecular dynamics and Monte Carlo simulations. It requires that there are a sufficient number of alchemical intermediate states especially if the end state perturbation is large. An alternative to FEP is thermodynamic integration. In thermodynamic integration a coupling parameter is introduced to define a series of non-physical intermediate states. The free energy change of two states is then calculated by integrating the derivative of potential energy over all coupling parameters [215]. The energy calculation methods employed in docking algorithms are fast and therefore useful in screening large databases of molecules. Rigorous free energy based methods are not suggested for screening large databases since they are much more time consuming. Even though energy calculations used in virtual high-throughput screening experiments can lead to the identification of hits, they are not reliable in predicting accurate binding affinities. Therefore they cannot be reliably used in lead optimization [112,217]. Recently, free energy calculation guided (FEP-guided) lead optimization has started to evolve [156]. The novel method BEDAM (binding energy distribution analysis method), based on statistical mechanics, is used to calculate binding free energies of target-ligand complexes [218]. BEDAM is an implicit solvent method that is implemented using Hamiltonian replica exchange molecular dynamics. Recently BEDAM showed success in the SAMPL4 (statistical assessment of the modeling of proteins and ligands) challenge in predicting free energies of binding for a set of octa-acid host–guest complexes [219]. VM2 is another method used in target–ligand binding energy calculations which falls between rigorous free energy calculation methods and approximate docking and scoring algorithms in its complexity [220]. It is an implicit solvent method and uses empirical force-fields. Its implementation is based on mining minima end point method (M2). In this method the binding site is taken to be fully flexible and the other parts of the target are kept fixed. Due to the flexibility of the binding site, it can adapt according to different bound ligands. The free energy is estimated to be the sum of all local energy minima.

#### Lead optimization and assessment of ADME and drug safety

When hits are obtained for a target structure by screening small molecule databases, the next step usually is lead optimization. During lead optimization, the effectiveness of promising hits obtained is generally enhanced while at the same time obtaining the desired pharmacological profiles to reach the required affinity, pharmacokinetic properties, drug safety, and ADME (absorption, distribution, metabolism, and excretion/elimination) properties. By increasing the affinity of a drug to the target its potency (efficacy) can be increased. The free energy of binding of a drug is a measurement of the potency of a drug to the target of interest. This could be done by doing alchemical free energy calculations in complex with running molecular dynamics simulations. One simulation starts with the target–ligand bound complex and slowly removes the ligand, and the other slowly removes the ligand from the solution. It is possible to find chemical changes of a possible drug candidate that can improve its potency using alchemical free energy calculations. This is done by gradually converting one atom of the ligand to another and calculating the binding affinity. These affinity changes with atom modifications can be used as guides for improving potency of drug candidates [194].

The permeability of a drug through the intestines and solubility are both important factors that affect drug absorption [221]. Therefore, in silico prediction of solubility and membrane permeability of drugs is an important part of lead optimization [222]. If an orally administrated drug has poor solubility or a high dissolution rate, the drug tends to be excreted by the body without entering the blood stream. This causes the drug to be inefficient and can even cause other biological side effects. To experimentally measure the solubility, the synthesis of the drug is needed which is a time consuming process. However, predicting solubility using computational methods is fast. It is possible to perform solubility calculations on large molecule libraries without needing a lot of computational resources. The solubility data can assist medicinal chemists to evaluate the drug candidates without having to synthesize molecules at all. This greatly reduces the costs of molecule synthesis and time for experimental solubility measurements. Huynh et al. used an in silico method for the prediction of solubility of docetaxel (DTX), an anti-cancer molecule used to treat various types of cancer [223]. In this study solubility parameters for DTX were obtained using MD simulations. This in silico model was in agreement with the experimental solubility of DTX. Simulation-based approaches are frequently used in computational permeability prediction [224–225]. In one study, trajectories obtained by molecular dynamic simulations have been used to obtain diffusion coefficients of permeation of drug-like molecules through the blood-brain barrier [225]. In silico approaches to predict drug solubility in both aqueous media and DMSO are discussed in a review [226].

Human intestinal absorption of a candidate drug is of high importance because it can affect the bioavailability of a drug. According to the Lipinski’s ‘Rule of 5’, poor absorption or permeation is more likely when: there are more than 10 H-bond acceptors, more than 5 H-bond donors, Log *P* is over 5, and the molecular weight is over 500 [227]. There are extensions of the Rule of 5 in predicting drug-likeliness as well [228]. One such extension later proposed is the ‘Rule of 3’ which was used in the construction of fragment libraries for lead generation [229]. These rules are generalized rules for evaluating the drug-likeness and bioavailability of compounds. Various statistical and mathematical models have been based on these rules and their extensions. Machine learning algorithms such as neural networks have been used in the prediction of drug-likeness and bioavailability [230–231].

QikProp is an ADME program offered by Schrodinger that predicts pharmaceutically relevant and physically significant descriptors for small drug-like molecules [232]. The VolSurf package can be used to calculate ADME properties and generate ADME models [233]. These ADME models can then be used to predict the behavior of novel molecules. It can also be used to find molecules with similar ADME properties as active ligands of interest. FAF-Drugs2 is an ADME and toxicity filtering tool that can calculate physicochemical properties, toxic and unstable groups, and key functional components [234]. Even though many possible drug molecules go to experimental verification stage or even animal models, they do not reach clinical trials. This is mostly due to the fact the drugs have poor pharmacokinetic properties and toxicity [235]. Thus filters for ADME properties are important for drug screening [236]. Computational ADME methods have advanced greatly in the last few decades and pharmaceutical companies are showing great interest in this area [237].

### Ligand-based drug design (LBDD)

The main alternative to SBDD is LBDD. In the case where the potential drug target structure is unknown and predicting this structure using methods such as homology modeling or ab initio structure prediction is challenging or undesirable, the alternative protocol to use is Ligand-based drug design [238–239]. Importantly, however, this method relies on the knowledge of small molecules that bind to the target of interest. Pharmacophore modeling, molecular similarity approaches and QSAR (quantitative structure–activity relationship) modeling are some popular LBDD approaches [240]. In molecular similarity methods, the molecular fingerprint of known ligands that bind to a target is used to find molecules with similar fingerprints through screening molecular libraries [241]. In ligand-based pharmacophore modeling, common structural features of ligands that bind to a target are used to do the screening [242]. QSAR is a computational method that models the relationship between structural features of ligands that bind to a target and the corresponding biological activity effect [243].

#### Similarity searches

The main idea of similarity-based or fingerprint-based approaches is to select novel compounds based on chemical and physical similarity to known drugs for the target. Ligand similarity search methods are simple but effective approaches based on the theory that structurally similar molecules tend to have similar binding properties [244]. These similarity measures do not take into account information about activities of known binders of the target. G-protein-coupled target GPR30 specific agonist that activates GPR30 was developed using similarity searches. The final similarity score that was used comprised a 2D score and a 3D structure similarity component [245–247].

#### Pharmacophore modeling

A pharmacophore is a molecular framework that defines the essential features responsible for the biological activity of a compound. When structural information about the drug target is limited or not known, pharmacophore models may be built using the structural characteristics of active ligands that bind to the target [248]. When 3D information of the target structure is known this binding site information can also be used in generating pharmacophore models [242]. Pharmacophore models that use chemical features such as acidic/basic residues and hydrogen bond acceptors and donors are found to be the most effective models [248]. Pharmacophore modeling has also been used in virtual screening of drugs in large databases [249]. There are programs developed to identify and generate pharmacophore models such as DISCO, GASP and Catalyst. It has been reported that GASP and Catalyst perform better than DISCO in reproducing the pharmacophore models [250]. One naturally occurring anti-cancer molecule identified using QSAR is I3C (indole-3-carbinol). However, this molecule has never gone past clinical trials due to its low potency. This active compound was optimized using ligand-based pharmacophore modeling to develop highly potent analog SR13668 which is a novel drug that shows to be highly potent against several cancer types [5]. Pharmacophore model construction steps can be summarized as follows:

The active compounds known to be binding to the desired target, that are also known to have the same interaction mechanism, are identified either by a literature search or a database search.
(a) For a 2D pharmacophore model essential atom types and their connectivity are defined (b) For a 3D pharmacophore model the conformations are defined using IUPAC nomenclature.
Ligand alignment or superimposition is used to find common features required in binders.
Pharmacophore model building.
Ranking of the pharmacophore models and selecting the best models.
Validation of pharmacophore models.

#### QSAR (quantitative structure–activity relationships)

QSAR methods are based on statistics that correlate activities of target drug interactions with various molecular descriptors. The basis of the QSAR method is the fact that structurally similar molecules tend to show similar biological activity [251]. These models describe mathematically how the activity response of a target, that binds a ligand, varies with the structural features of the ligand. QSAR is obtained by calculating the correlation between experimentally determined biological activity and various properties of small ligand binders [243]. QSAR relationships can be used to predict the activity of new drug molecule analogs.

In order to quantify the activity of a drug molecule, several values can be used. Half maximal inhibitory concentration (IC_50_) and inhibition constant (*K*_i_) are the most commonly used measures. QSAR models, unlike the pharmacophore models, can be used to find the positive or negative effect of a particular feature of a drug molecule to its activity. QSAR methods have been used successfully on various drug targets such as carbonic anhydrase [252–253], thrombin [254–255] and renin [256]. Different machine learning techniques have also been used in constructing QSAR models [257–259]. In classical or 2D QSAR methods, the biological activity is correlated to physical and chemical properties such as electronic hydrophobic and steric features of compounds [260]. In more advanced 3D QSAR methods, in addition to physical and geometric features of active drug molecules, quantum chemical features are also used. Recently QSAR models have also been developed for membrane systems [261].

**Figure 8:**
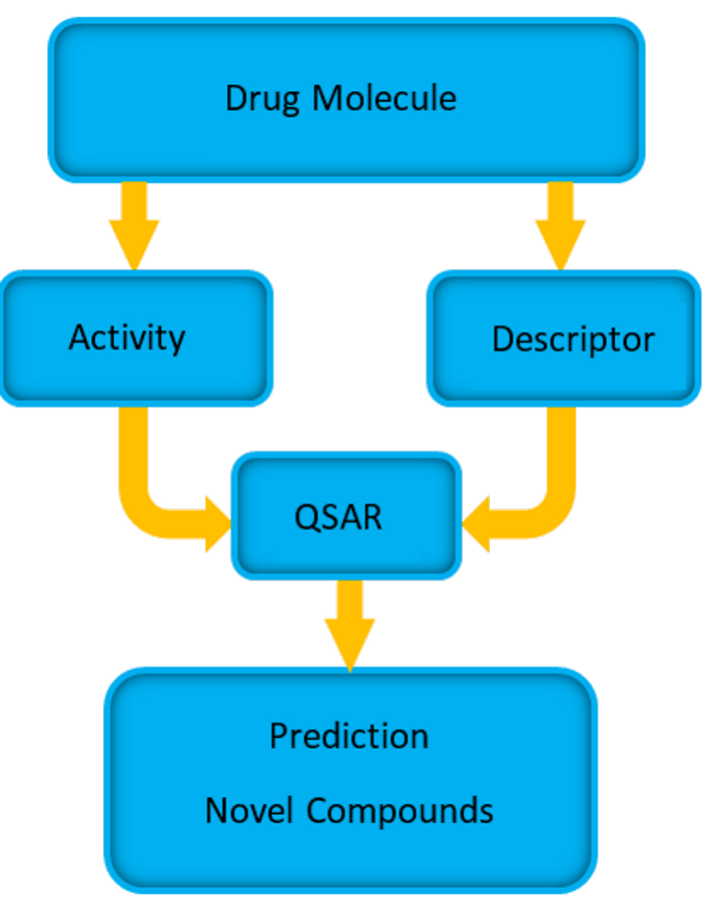
Schematic diagram showing the steps involved in QSAR. Known drug molecule activity and descriptor data is obtained and the mathematical model of QSAR is built such that descriptors can predict the activity of each molecule. The predictive power of models are validated and used in predicting activities of novel compounds.

The basic steps (Figure 8) of the QSAR method can be summarized as follows:

1. The active molecules that bind to the desired drug target and their activities are identified through a database search, a literature search, or HTS experiments.
2. Identification of structural or physicochemical molecular features (fingerprint) affecting biological activity (e.g. bond, atom, functional group counts, surface area etc.).
3. Building of a QSAR between the biological activity and the identified features of the drug molecules.
4. Validation of the QSAR biological activity predictive power.
5. Use of the QSAR model to optimize the known active compounds to maximize the biological activity.
6. The new optimized drug molecule activities are tested experimentally.

Success of a QSAR depends on the molecular descriptors selected and the ability of these models to predict biological activity. If there is not enough activity data to extract patterns, QSARs cannot perform well. Therefore, this method requires a certain minimum amount of training data in order to build a good predictive model and it is often linked to high-throughput screening. Statistical methods have been used in linear QSAR to pick molecular descriptors that are important in predicting the biological activity. MLR (multivariable linear regression) can be used to find molecular descriptors that have a good correlation with the target–ligand biological activity. It is only possible to use linear regression methods if the activity descriptor relation is linear. However the relationship between biological activity and the molecular descriptors are not always linear [262]. Machine learning approaches such as neural networks and support vector machine methods are used to generate QSAR models to address this issue of non-linear fitting [263–265]. Principal component analysis (PCA) can be used to simplify the complexity by removing the descriptors that are not independent [266]. Once the right set of features is identified and the QSAR is built, these models can be validated using methods such as cross validation [267–268]. QSAR models can be used to predict the biological activity of novel molecules by just using the molecular features. Thus these models can be used to screen a database of molecules to find potential active molecules.

**Figure 9:**
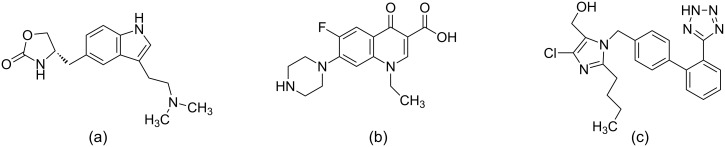
A few drugs discovered with the help of ligand-based drug discovery tools. (a) Zolmitriptan: used as a treatment to migraine (b) Norfloxacin: used in urinary tract infections and (c) Losartan: used to treat hypertension.

Some of the drugs that are on the market with the help of ligand-based drug discovery are Zolmitriptan, Norfloxacin and Losartan [8]. Norfloxacin is a drug that is used in urinary tract infections and was developed using a QSAR model and approved by the FDA in 1986 [269]. Losartan [270] is used to treat hypertension and Zolmitriptan [271] is used as a treatment to migraine (Figure 9).

One difference between pharmacophore models and QSAR is that the pharmacophore model is constructed based on the necessary or essential features of an active ligand, whereas QSAR takes into account not only the essential features but also the features that affect the activity. One important structural feature used in both the pharmacophore model and in QSAR is the volume of the binding site. It is well established that the binding pocket volume has a big influence on the biological activity. In the cases where the binding pocket volume is known, elimination of molecules that are too large to fit in the binding pocket can be done in early stages of drug discovery process (see section “Binding pocket identification and volume calculation”).

#### Role of machine learning in LBDD

Machine learning algorithms can be trained to identify patterns in data and used to do predictions on test data sets. These algorithms are extensively applied in the field of biology and drug discovery [272–275]. Machine learning is used in many stages in the drug discovery pipeline including in the QSAR analysis stage [276]. Support vector machine (SVM) based algorithms are commonly used and have been shown to have high predictive power. SVM are often used for classification of sets of biological data. For example, they can be used to distinguish between molecules that have high affinity for a target and those that have no affinity. Machine learning based scoring functions can also be used in structure-based drug discovery to predict target–ligand interactions and binding affinities [277]. Compared to conventional scoring functions, machine learning based scoring functions have often shown comparable or even improved performance. Moreover these algorithms can be trained to distinguish active drugs from decoys that do not have known drug activity [278]. Artificial neural networks (ANNs) have been used in drug discovery as a powerful predictive tool for non-linear systems [279]. For example, ANNs were used to construct the QSAR of a set of known aldose reductase inhibitors and biological activities of new molecules were predicted based on the QSAR [280]. Docking algorithms were then used to find novel inhibitors that bind to aldose reductase. ANN-based prediction models are also used in predicting biotoxicity of molecules as well [281].

## Conclusion

In the past 10 years the identification rate of disease-associated targets has been higher than the therapeutics identification rate. With considerable rise in the number of drug targets, computational methods such as protein structure prediction methods, virtual high-throughput screening and docking methods have been used to accelerate the drug discovery process, and are routinely used in academia and in the pharmaceutical industry. These methods are well established and are now a valuable integral part of the drug discovery pipeline and have shown great promise and success. It is cheaper and faster to computationally predict and filter large molecular databases and to select the most promising molecules to be optimized. Only the molecules predicted to have the desired biological activity will be screened in vitro. This saves money and time because the risk of committing resources on possibly unsuccessful compounds that would otherwise be tested in vitro is reduced.

Structure-based and ligand-based virtual screening methods are popular with most of the applications being directed towards enzyme targets [282]. Even though structure-based methods are more frequently used, ligand-based methods have led to the discovery of an impressive number of potent drugs. In SBDD knowing the three-dimensional structure of the target of interest is required. However, in some cases it is not possible to determine structures of targets using conventional experimental methods due to experimental challenges. In the cases where experimental methods fail, computational methods become useful and potentially necessary for SBDD [23]. In the absence of an experimentally determined structure or a computationally generated model for a target of interest LBDD tools can be used. These tools require the knowledge of active drugs that bind to the target. LBDD tools such as 2D and 3D similarity searches, QSAR and pharmacophore modeling have proven successful in lead discovery.

Experimental methods usually represent proteins as static structures. However proteins are highly dynamic in character and protein dynamics play an important role in their functions. Computational modeling of the flexible nature of proteins is of great interest and various ensemble-based methods in structure-based drug discovery have emerged [178]. Molecular dynamics simulations are widely used in generating target ensembles that can be subsequently used in molecular docking [178]. Docking tools have been developed with different scoring functions and search algorithms. Comparative studies have been performed to evaluate these scoring functions and docking algorithms in docking pose selection and virtual screening [112,144,283]. There is no one superior tool that works for all target–ligand systems. The quality of docking results is highly dependent on the ligand and the binding site of interest [148–150].

VHTS methods are useful to screen large small molecule repositories fast and pick a smaller number of possible drug-like molecules for testing. By reducing the number of possible molecules that need to be tested experimentally, these methods can help to greatly cut the cost associated with drug discovery process. Studies have shown that with VHTS it is possible to identify molecules that are not observed with conventional high-throughput screening (HTS) experiments [284]. Thus VHTS methods are frequently used to complement HTS methods. The molecules selected by both of these methods are more likely to be possible drug candidates and should be considered when selecting hits.

Absorption, distribution, metabolism and elimination/excretion properties, commonly abbreviated ADME, as well as toxicity are important for the ultimate success or failure of a possible drug candidate. Adverse effects in animal models or even clinical trials can be reduced by filtering drug candidates by their ADME properties in early stages. Another important fact to consider in drug safety studies is how one drug can affect the metabolic stability of another drug [285]. Some drug–drug interactions (DDI) could lead to serious health effects; therefore, predicting these effects is important but challenging. The prescription antihistamine terfenadine and antifungal drug ketoconazole are two examples of drugs that should not be co-administered [286]. Terfenadine–ketoconazole drug–drug interactions results in cardiotoxicity. Computational methods such as pharmacokinetic modeling and predicting drug–drug interactions using large DDI interaction databases are successful and are both cost and time saving as well [287–288].

Currently hybrid structure-based and ligand-based methods are also gaining popularity. These combined (ligand-based and structure-based) drug discovery methods are of interest because they can amplify the advantages of both methods and improve the protocols [289–291]. One example is the hybrid docking protocol HybridDock, which incorporates both structure-based and ligand-based methods [291]. This hybrid method shows significantly improved performance in both binding pose and binding affinity prediction.

CADD has had a significant impact on the discovery of various therapeutics that are currently helping treat patients. Despite the successes, CADD also faces challenges such as accurate identification and prediction of ligand binding modes and affinities [8]. One of the challenging areas in drug discovery is the phenomenon of drug polymorphism [292]. Drug polymorphism occurs when a drug has different forms which are identical chemically but differ structurally. This can have a great impact on the success of a drug. Different polymorphic forms of a drug, which have different solid-state structures, can differ in solubility, stability and dissolution rates. Drug polymorphism can affect the bioavailability, efficacy and toxicity of a drug. One polymorphic form that is responsible for a particular drug effect may differ if a different polymorphic form of the same drug is administered. Using techniques such as spectroscopy it is possible to characterize drugs having different polymorphic forms.

Protein–protein interactions (PPIs) pose another challenge in drug discovery. PPIs are involved in many cellular processes and biological functions that are linked to diseases. Therefore, small molecule drugs that aim at PPIs are important in drug discovery [293]. It is of interest to develop therapeutics that can either disrupt or stabilize these interactions. However, it is challenging to design inhibitors that can directly interrupt PPIs. Common drug design usually targets a specific binding site on a protein of interest. However, protein–protein interacting surfaces have larger interfaces and are more exposed. Therefore their binding sites are often not well defined. Finding the sites that can be aimed at in PPI inhibition is therefore challenging and of great importance.

Through collaboration with the Drug Design Data Resource (D3R), which is a project funded by the National Institutes for Health, pharmaceutical companies are able to release their previously unreleased drug discovery data to the scientific community. This project allows the scientists all over the world to use new high-quality data in improving computer-aided drug discovery and design, and also to speed up the progress. The field of CADD is continuously evolving with improvements being made in each and every area. Some of the focus areas are scoring functions, search algorithms for molecular docking and virtual screening, optimization of hits, and assessment of ADME properties of possible drug candidates. With the current successes there is a promising future for computational methods to aid in the discovery of many more therapeutics in the future.
